# Analysis of genetic polymorphisms for age-related macular degeneration (AMD) in Chinese Tujia ethnic minority group

**DOI:** 10.1186/s12881-019-0756-4

**Published:** 2019-01-29

**Authors:** Shengchun Liu, Mingxing Wu, Bianwen Zhang, Xiaojing Xiong, Hao Wang, Xiyuan Zhou

**Affiliations:** 1 Department of Ophthalmology, the Second Affiliated Hospital of Chongqing Medical University, NO.74, Linjiang Road, Yuzhong District, Chongqing, 400010 China; 2 Chongqing Key Laboratory of Ophthalmology and Chongqing Eye Institute, Chongqing, 400010 China

**Keywords:** Age-related macular degeneration, Single nucleotide polymorphism, Chinese Tujia ethnic minority group

## Abstract

**Background:**

Age-related macular degeneration (AMD) can cause vision loss or blindness in elderly. The associations between single nucleotide polymorphism (SNP) and AMD in Chinese Tujia ethnic minority group are still unclear.

**Methods:**

A total of 2122 Tujia volunteers were recruited and 197 of them were diagnosed with AMD (either dry or wet type).Then the blood specimens of these 197 AMD patients and 404 controls from the remaining 1925 normal Tujia volunteers were collected to detect the frequencies of 39 chosen SNPs. The Bonferroni method was used to correct the *P* values from the Fisher’s exact test.

**Results:**

The mean age of the 197 AMD patients(113 males and 84 females) was 68.4197 years old. No significant differences in allelic and genotypic frequencies were found for all the 39 SNPs between the patients and controls. However, weak correlations between 10 SNPs (CFH rs1329428 TT genotype, CFH rs3753394 CC genotype and T allele, CFH rs1410996 AA genotype, CFH rs800292 AA genotype, CFH rs800292 A allele, VEGF rs833061 TT genotype and C allele, VEGF rs2010963 CG genotype, VEGFR2 rs1531289 TT genotype, ARMS2 rs10490924 TT genotype, KCTD10 rs238104 GC genotype, rs1531289 T allele and ARMS2 rs10490924 T allele) and AMD were shown.

**Conclusions:**

The effects of 39 SNPs have found no associations with the morbidity of AMD in Chinese Tujia ethnic minority group.

**Electronic supplementary material:**

The online version of this article (10.1186/s12881-019-0756-4) contains supplementary material, which is available to authorized users.

## Background

Age-related macular degeneration (AMD) is the main cause of blindness and vision loss in old people in developed countries [1]. The formation of deposits, inflammation and ultimately neurodegeneration in the macula are typical features of the disease. In general, AMD can be divided into two subtypes: the non-exudative (dry or atropic) subtype and the exudative (wet or neovascular) subtype [2]. The development of the disease is a complex interplay of age, environmental, genetic, metabolic and many other factors [3].

Epidemiological and gene-mapping studies supported that the genetic factors played important roles in the pathogenesis of AMD [4, 5]. A genome-wide study reported 52 independently AMD associated variants by analyzing more than 15,000 patients and controls. The results also showed that rare variants could directly affect causal genes [6]. In addition, other researchers found that the loci 3p13 and 10q26 had a relationship with the complex basis of the AMD by analyzing 70 families and these genes were involved in immune response, inflammation and retina homeostasis [7]. Genome-wide association study had also been used to clarify the possible relationships between SNPs and outcomes of anti- vascular endothelial growth factor (VEGF) treatment for exudative AMD and the results showed that the age-related maculopathy susceptibility 2 (ARMS2/HTRA1) polymorphism rs10490924 might be a good marker to predict the effect of ranibizumab treatment [8].

The Chinese Tujia ethnic minority group mainly live on th mountains in the middle of China. Due to the relative isolation of mountain, this ethnic minority group may have its own specific genome. The genetic analysis of Y chromosome in Chinese Tujia ethnic minority group demonstrated that the 17 Y-STR loci were highly polymorphic [9]. Previous studies conducted by Chinese epidemiologists reported that gene variants in CFH, ARMS2 and HTRA1 were related to an increased risk of AMD in a northern Chinese population, which was partially consistent with the results of the western world [10, 11]. However, the epidemiology analysis of AMD in Chinese Tujia ethnic minority group and its potential pathogenic mechanism had not been reported.

In this study, we calculated the morbidity of AMD in 2122 Tujia volunteers. Then we analyzed the frequencies of 39 AMD-associated SNPs in 197 AMD patients and 404 normal controls. Our goal was to identify the possible pathogenic SNPs of AMD in Chinese Tujia ethnic minority group.

## Methods

### Patients and data collection

Our study recruited 2122 individuals who belonged to Chinese Tujia ethnic minority group in the Second Affiliated Hospital of Chongqing Medical University from January 2009 to December 2016 (Chongqing, China). Diagnosis and grading for AMD followed the standard of clinical age-related maculopathy staging (CARMS) system and maculopathy could be classified into five grades. Grade 1: no drusen or 10 small drusen without pigment abnormalities. Grade 2: approximately 10 small drusen or 15 intermediate drusen. Grade 3: approximately 15 intermediate drusen or any large drusen. Grade 4: geographic atrophy with involvement of the macular center. Grade 5: exudative AMD. Volunteers with Grade 2 or above AMD (either unilateral eye or bilateral eyes) were recruited as the patients group [12]. The ethics committee of the Second Affiliated Hospital of Chongqing Medical University approved the study and the medical records and blood samples were obtained from volunteers with written informed consents.

### Single nucleotide polymorphism (SNP) selection

Target genes and SNPs were chosen according to previously published studies [13–26]. As a result, 39 SNPs of 16 genes were selected.and included 8 SNPs (rs1061170, rs800292, rs3753394, rs1410996, rs1329428, rs6677604, rs380390, rs10737680) of complement factor H(CFH), 2 SNPs (rs4151667, rs641153) of complement factor B (CFB), 2 SNPs (rs9332739, rs547154) of complement C2(C2), 1 SNP (rs2241394) of complement C3(C3), 1 SNP (rs2511989) of serpin family G member 1(SERPING1), 1 SNP (rs10490924) of ARMS2, 7 SNPs (rs10033900, rsl3117504, rs11726949, rs6854876, rs11728699, rs7439493, rs4698775) of complement factor I(CFI), 2 SNPs (rs3732379, rs3732378) of C-X3-C motif chemokine receptor 1(CX3CR1), 4 SNPs (rs943080, rs3025039, rs833061, rs2010963) of VEGF, 4 SNPs (rs9554322, rs7337610, rs9582036, rs9943922) of vascular endothelial growth factor receptor (VEGFR), 1 SNP (rs1531289) of VEGFR2, 1 SNP (rs6987702) of tribbles pseudokinase 1(TRIB1),2 SNPs (rs1800775, rs3764261) of cholesteryl ester transfer protein(CETP), 1 SNP (rs2338104)of potassium channel tetramerization domain containing 10(KCTD10/MVK),1 SNP (rs8017304) of RAD51 paralog B (RAD51B) and 1 SNP (rs1883025) of ATP binding cassette subfamily A member 1(ABCA1).

### DNA extraction and genotyping

Peripheral blood of AMD patients and the controls were subjected to genomic DNA extraction by using the QIAmp DNA Blood Mini Kit (Qiagen Inc., Valencia, CA, USA) and the DNAs were stored at − 80∘C. Genotype identifications of the 39 SNPs were conducted with the iPLEX Gold genotyping assay and Sequenom MassARRAY (Sequenom, CA, USA). Sequenom SNP Assay Design software (version 3.0) was used to design the primers of iPLEX reactions [27]. Primer sequences used were shown in Additional file 1: Table S1.

### Statistical analysis

Hardy-Weinberg equilibrium (HWE) analysis was carried out in normal controls and no SNPs significantly deviated from HWE (*P* > 0.05). Fisher’s exact test was applied to evaluate the differences in allele and genotype frequencies of all SNPs between patients and healthy controls by using SPSS (version 19.0; SPSS Inc., Chicago, IL). The Bonferroni method was conducted to perform correction for multiple comparisons whereby the *P* value was multiplied with the number of comparisons (*P* corrected (*Pc*)) [27]. It was considered to be significant when *Pc* was less than 0.05.

## Results

**Fig. 1:**
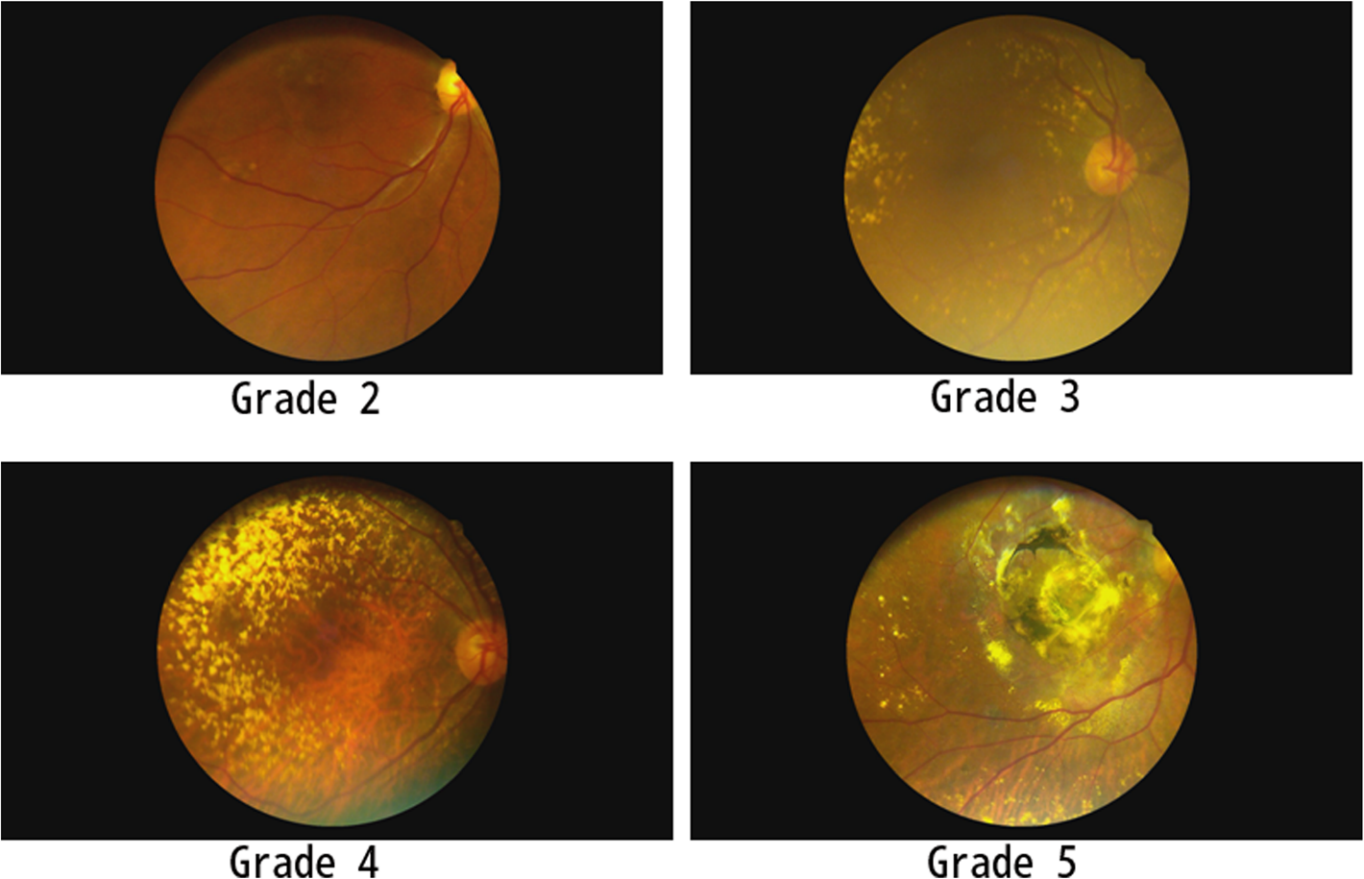
the representative images of grade 2 to grade 5 AMDs from our patients

A total of 2122 volunteers aged from 50 to 90 years old were recruited to our study. The fundus examination was used to diagnose and divided the volunteers into five grades according to the clinical age-related maculopathy staging (CARMS) system. The representative images of grade 2 to 5 AMD were shown in Fig. 1. Among the 2122 volunteers, we found that 197 cases (113 males and 84 females) could be diagnosed as AMD and the mean age was 68.4ales)we foundAM (Table 1). Moreover, only 404 normal volunteers (245 males and 159 females, mean age was 63.5 ± 04 normal volu) accepted the SNPs examinations and we assigned them to the normal control group.

**Table 1:** The age and grade distribution of AMD patients

Age	Number(%)	AMD				
		Total	Grade 2	Grade 3	Grade 4	Grade 5
50–59	456(21.49%)	25(5.48%)	16	7	0	2
60–69	965(45.48%)	95(9.84%)	57	37	0	1
70–79	599(28.23%)	68(11.35%)	37	26	4	1
80-	102(4.81%)	9(8.82%)	5	3	1	0
Total	2122	197	115	73	5	4

Next, the blood specimens from AMD cases and controls were collected to detect the genome sequences. We chose 39 SNPs covering 16 genes to figure out if these SNPs could be pathogenic factors for AMD in Chinese Tujia ethnic minority group. As a result, we found that all 39 SNPs of controls met the Hardy-Weinberg equilibrium. No significant differences in both allelic and genotypic frequencies were found for all the 39 SNPs between the patient and control groups according to the P_c_ values (Additional file 2: Table S2). However, the *P* values showed weak correlations between 10 SNPs of 5 genes and AMD (Table 2). Compared with the AMD patients, the frequencies of the CFH rs1329428 TT genotype (*P* = 0.023, OR = 1.649 and 95% CI = 1.069–2.543), CFH rs3753394 CC genotype(*P* = 0.006, OR = 1.738 and 95% CI = 1.164–2.594) and T allele(*P* = 0.029, OR = 1.307 and 95% CI = 1.027–1.664), CFH rs1410996 AA genotype(*P* = 0.008, OR = 1.814 and 95% CI = 1.164–2.826) and CFH rs800292 AA genotype(*P* = 0.009, OR = 1.787 and 95% CI = 1.154–2.769) were decreased in the controls. On the contrary, the frequency of the CFH rs800292 A allele (*P* = 0.011, OR = 0.730 and 95% CI = 0.571–0.932) was increased in the controls. Moreover, the frequencies of the VEGF rs833061 TT genotype (*P* = 0.020, OR = 1.511 and 95% CI = 1.067–2.138) and C allele (*P* = 0.021, OR = 1.390 and 95% CI = 1.051–1.837), VEGF rs2010963 CG genotype(*P* = 0.032, OR = 1.462 and 95% CI = 1.033–2.071), VEGFR2 rs1531289 TT genotype (*P* = 0.040, OR = 2.025 and 95% CI = 1.020–4.022), ARMS2 rs10490924 TT genotype(*P* = 0.002, OR = 1.928 and 95% CI = 1.280–2.904) and KCTD10 rs238104 GC genotype (*P* = 0.019, OR = 1.505 and 95% CI = 1.068–2.120) were decreased and the frequencies of VEGFR2 rs1531289 T allele (*P* = 0.012, OR = 0.690 and 95% CI = 0.516–0.924)and ARMS2 rs10490924 T allele (*P* = 0.037, OR = 0.687 and 95% CI = 0.482–0.978) were increased in the controls comparing with the AMD patients.

**Table 2:** Genotype and allele frequencies of ten genes’ polymorphism in AMD and healthy controls

Genes	SNPs		Case	Control	HWE	P	Pc	OR	95%Cl
CFH	rs1329428	Total sample	197	404					
		CC	59	136	0.175	0.361	NS	0.842	0.583–1.217
		CT	94	208		0.386	NS	0.860	0.612–1.209
		TT	44	60		0.023	NS	1.649	1.069–2.543
		C	212	480					
		T	182	328		0.065	NS	0.796	0.624–1.015
CFH	rs3753394	Total sample	197	403					
		CC	55	74	0.416	0.006	NS	1.738	1.164–2.594
		CT	89	208		0.163	NS	0.784	0.558–1.104
		TT	53	124		0.357	NS	0.837	0.573–1.223
		C	199	356					
		T	195	456		0.029	NS	1.307	1.027–1.664
CFH	rs1410996	Total sample	190	386					
		GG	60	137	0.128	0.469	NS	0.873	0.603–1.262
		GA	87	204		0.194	NS	0.795	0.562–1.125
		AA	43	55		0.008	NS	1.814	1.164–2.826
		G	207	478					
		A	173	314		0.056	NS	0.786	0.614–1.006
CFH	rs800292	Total sample	193	402					
		GG	54	140	0.190	0.095	NS	0.727	0.500–1.058
		GA	95	205		0.686	NS	0.932	0.661–1.313
		AA	44	57		0.009	NS	1.787	1.154–2.769
		G	203	485					
		A	183	319		0.011	NS	0.730	0.571–0.932
VEGF	rs833061	Total sample	192	403					
		TT	113	196	0.881	0.020	NS	1.511	1.067–2.138
		TC	67	171		0.079	NS	0.727	0.509–1.039
		CC	12	36		0.261	NS	0.680	0.345–1.338
		T	293	563					
		C	91	243		0.021	NS	1.390	1.051–1.837
VEGF	rs2010963	Total sample	189	396					
		CC	27	64	0.089	0.558	NS	0.865	0.531–1.408
		CG	99	170		0.032	NS	1.462	1.033–2.071
		GG	63	162		0.078	NS	0.722	0.502–1.038
		C	153	298					
		G	225	494		0.349	NS	1.127	0.877–1.448
VEGFR2	rs1531289	Total sample	197	404					
		CC	118	275	0.181	0.048	NS	0.701	0.492–0.998
		CT	62	111		0.310	NS	1.212	0.836–1.758
		TT	17	18		0.040	NS	2.025	1.020–4.022
		C	298	661					
		T	96	147		0.012	NS	0.690	0.516–0.924
ARMS2	rs10490924	Total sample	197	404					
		GG	57	137	0.625	0.213	NS	0.791	0.546–1.145
		GT	86	200		0.169	NS	0.786	0.558–1.108
		TT	54	66		0.002	NS	1.928	1.280–2.904
		G	200	474					
		T	194	332		0.008	NS	0.722	0.567–0.920
KCTD10/MVK	rs2338104	Total sample	197	403					
		GG	19	44	0.673	0.633	NS	0.871	0.494–1.536
		GC	110	184		0.019	NS	1.505	1.068–2.120
		CC	68	175		0.037	NS	0.687	0.482–0.978
		G	148	272					
		C	246	534		0.193	NS	1.181	0.919–1.518
CX3CR1	rs3732378	Total sample	193	402					
		AA	3	0	0.573	0.034	NS		
		AG	12	22		0.714	NS	1.145	0.554–2.365
		GG	178	380		0.277	NS	0.687	0.348–1.356
		A	18	22					
		G	368	782		0.084	NS	1.739	0.921–3.281

## Discussion

In present study, we compared the frequencies of 39 SNPs of 16 genes between 193 AMD patients and 404 controls from Chinese Tujia ethnic minority group. It had reported that ARMS2 and CFH variants were associated with neovascular AMD in the Thai, Korean and Chinese Han population [28–30] and no previous studies focused on the associations between SNPs and AMD in Tujia ethnic minority group. Therefore, we designed this research to identify the potential associations. Finally, our results showed that no significant differences for these 39 SNPs were found between the two groups. However, the *P* value suggested that the AMD had weak correlations with CFH SNPs, VEGF family SNPs and ARMS2 SNP.

The major candidate genes for AMD pathogenesis were CFH and ARMS2 [31, 32]. Previous study reported gene variants in CFH and ARMS2 were related to increased risks of AMD in Chinese Han population [33]. However, our results showed negative correlation, which might be caused by the racial and sample size differences. Furthermore, VEGF gene played an important role in regulating angiogenesis and permeability [34]. The SNPs of VEGF were related to the formation of choroidal neovascularization in exudative AMD. Therefore, anti-VEGF agents had been widely used to treat the exudative AMD. The alleles in CFH, ARMS2, and VEGFA were associated with genetic anticipation and inadequate response to the anti-VEGF agents in AMD patients [35]. The relationship between the delayed functional and limited response to the injection of bevacizumab and the CFH gene polymorphism T1277C was also identified [36]. In our study, no associations were found between the SNPs of VEGF family genes and the morbidity of AMD. However, a stratified analysis had not been carried out and the relationships between SNPs of VEGF family genes and morbidity of exudative AMD were still unclear. In addition, the SNPs of VEGF family genes might affect the AMD by impacting the drug responses in Chinese Tujia ethnic minority group.

Our study had several limitations. We only chose SNPs that have been previously reported and no new SNPs were found. A genome-wide study should be carried out to find more pathogenic SNPs. Furthermore, the stratified analysis of different ages, genders or AMD types should also been used to deeply investigate the associations between the SNPs and the AMD morbidity in Chinese Tujia ethnic minority group. Last, we only recruited a very small sample size of patients in our study and the representativeness of our findings was limited. In the future, we would collect more patients to perform the SNP detections.

## Conclusions

In sum, the chosen 39 SNPs had no associations with the morbidity of AMD in Chinese Tujia ethnic minority group.

## Additional files

Additional file 1: **Table S1.** Primer sequences we used to detect the 39 SNPs were listed in the table. (XLSX 11 kb)

Additional file 2: **Table S2.** The distributions of allelic and genotypic frequencies for 39 SNPs were listed in the table. The details of HWE, *P* value, P_correct_ value, OR and 95%CI were also shown. (XLS 102 kb)

## Abbreviations

AMD: Age-related macular degeneration
CARMS: Clinical age-related maculopathy staging
HWE: Hardy-Weinberg equilibrium
SNP: Single nucleotide polymorphism
